# Digital Tools and Lifestyle-Based Dementia Risk Reduction: Lessons From World-Wide FINGERS

**DOI:** 10.1093/geroni/igaf122.1142

**Published:** 2025-12-31

**Authors:** Su-I Hou, Parthenia Giannakopoulou, Miia Kivipelto, Francesca Mangialasche, Paola Padilla, Alina Solomon

**Affiliations:** University of Central Florida, Orlando, Florida, United States; Imperial College London, London, England, United Kingdom; Karolinska Institutet, Solna, Stockholms Lan, Sweden; Division of Clinical Geriatrics, Stockholm, Stockholms Lan, Sweden; FINGERS Brain Health Institute, Stockholm, Stockholms Lan, Sweden; Karolinska Institutet, Solna, Stockholms Lan, Sweden

## Abstract

**Introduction:**

The Finnish Geriatric Intervention Study to Prevent Cognitive Impairment and Disability (FINGER) was the first large, long-term randomized controlled trial to show that a two-year multidomain lifestyle intervention improved cognitive function in older adults at risk for dementia. Computer-based cognitive training was a key component. The FINGER model has been globally adapted through the World-Wide FINGERS (WW-FINGERS) network, officially launched in 2017, with growing use of digital tools.

**Methods:**

WW-FINGERS, the first global network of multimodal dementia prevention trials, includes research teams from 70 countries. We reviewed trial protocols and published results from the WW-FINGERS Global Scientific Coordinating Center, identifying 20 ongoing and 15 completed trials, including pilot studies.

**Results:**

Digital tools have been integrated into seven trials: Maintain Your Brain (MYB, Australia), FINGER (Finland), MIND-ADmini (multi-country), AgeWell.de (Germany), J-MINT (Japan), MIND-CHINA (China), and SUPERBRAIN (South Korea). Nineteen published protocols and results were analyzed. Study populations ranged from dementia-free older adults with risk factors or mild cognitive impairment. Digital tools were primarily used for cognitive training via computer programs, web platforms, or tablet applications. Some trials used wearable devices to monitor physical activity. However, discussion on digital tool implementation was limited. MYB was the first fully internet-based WW-FINGERS study, demonstrating online lifestyle interventions can improve cognition and health outcomes.

**Discussion:**

Digital tools are feasible in dementia prevention interventions across diverse populations. Their adoption has increased since the original FINGER study started in 2009. Selection should consider participants’ cognitive abilities, and social engagement components may enhance usability.

